# Controversies and Challenges of Mass Vaccination against SARS-CoV-2 in Italy: Medico-Legal Perspectives and Considerations

**DOI:** 10.3390/healthcare9091163

**Published:** 2021-09-05

**Authors:** Rosario Barranco, Gabriele Rocca, Andrea Molinelli, Francesco Ventura

**Affiliations:** Department of Legal and Forensic Medicine, University of Genova, Via De’ Toni 12, 16132 Genova, Italy; gabriele.rocca@unige.it (G.R.); a.molinelli@unige.it (A.M.); francesco.ventura@unige.it (F.V.)

**Keywords:** COVID-19, legal medicine, vaccine, professional liability

## Abstract

The worldwide spread of SARS-CoV-2 and the pandemic has resulted in a serious global crisis in the health, social and economic spheres. After an initial period of enthusiasm related to the efficacy of vaccines, in many European countries, a growing distrust in the population has matured, due to the reporting of severe adverse effects. Throughout the world, some cases of adverse events after the administration of the vaccine have been reported. In this communication, we want to discuss all the medico-legal aspects related to the global vaccination companion in terms of medical professional responsibility, informed consent and vaccination obligation, with particular attention to the Italian situation. Health professionals are tasked with promoting confidence in vaccination for the general population. Complete and detailed information and reliance on scientific research is essential to understand the great importance of the vaccination campaign. From a criminal point of view, we must avoid blaming health professionals in the case of side effects. At the same time, we must protect the population, ensuring compliance with the indications, guidelines, and an adequate method of administration. On the other hand, from a civil law perspective, it is correct to ensure full protection of those rare cases in which the administration of the vaccine is related to adverse events. Without a broad and global vaccination campaign, it will be impossible to overcome COVID-19.

## 1. Introduction

The worldwide spread of SARS-CoV-2 and the pandemic has led to a serious global crisis in the health, social and economic spheres. Throughout the world, various governments have implemented containment measures and social barriers to reduce contagion from COVID-19, such as the use of face masks, social distancing, generalized lockdowns and movement restrictions [1,2]. The hope of winning the global battle against COVID-19 was given by the approvals of vaccines, capable of preventing the SARS-CoV-2 disease. At present, four different vaccines are available in Europe that use different technologies. In particular, the Pfizer–BioNTech vaccine was approved on 21 December 2020 [3,4]; the Moderna mRNA-1273 vaccine was approved on 6 January 2021 [3,5]; the third vaccine, ChAdOx1 nCov-19 (AstraZeneca), was authorized on 29 January 2021 [3,6]; and the latest vaccine is COVID-19 Vaccine Janssen (Johnson & Johnson), which was declared valid on 11 March 2021 [3,7]. Furthermore, other vaccines are currently undergoing clinical trials and trials [3,8].

Surely, rapid large-scale vaccination of the world’s population represents the best way to restore normal life and global economies [9].

After an initial period of enthusiasm related to the efficacy of vaccines, in many European countries, a growing distrust in the population has matured due to the reporting of severe adverse effects. In fact, in April 2021, the European Medicines Agency stated that unusual blood clots with low blood platelets could be rare side effects of the Astrazeneca vaccine [10]. Many scientific studies have analyzed the correlation between thrombosis/thrombocytopenia and ChAdOx1 nCoV-19 vaccination [3,11,12,13]. The pathophysiological mechanism is not yet well known. It is possible the formation of antibodies against PF4 (heparin-induced thrombocytopenia platelet factor 4 antibody) that cause the consumption of platelets with low platelet counts and thrombus formation [12]. It was also confirmed that very small numbers of vascular adverse events (blood clots in combination with low platelets) can occur within approximately one to three weeks after Janssen vaccination [3,14].

Furthermore, after the start of the vaccination campaign with BioNTech/Pfizer, anaphylactic reactions and shock have been reported [15].

In any case, the incidence of post-vaccination adverse effects is extremely low, and the benefit/risk ratio of vaccines remains high, especially in adults and the elderly [16].

The distrust of vaccines and the development of “no-vax” currents have taken root among the population, despite the assurances of doctors and the serious risks related to the contagion and development of SARS-CoV-2 disease [1,17]. This was also due to an imprecise information campaign, which tended to exalt the possible adverse effects.

There is no doubt that any drug can cause adverse reactions, but we must keep in mind the risk/benefit ratio and consider the interests of the community. Risk 0 in the medical and health sector does not exist in any case.

From this complex situation, possible medical–legal and ethical debates emerge, as occurred during the current pandemic [1,18].

In this short communication, we want to discuss all the medico-legal aspects related to the global vaccination campaign in Italy, in terms of medical professional responsibility, informed consent and mandatory vaccination.

## 2. Medical Liability Related to Vaccination: Criminal Aspects

When the alarm about the potential side effects of AstraZeneca’s COVID-19 vaccine stopped vaccinations across Europe in March 2021, there was a growing distrust of vaccines [19]. Although the greatest attention has been paid to the Astrazeneca vaccine, serious adverse reactions have been reported after administration of all commercial vaccine types [20].

As of 26 June 2021, in Italy, the reporting rates of serious adverse events of individual vaccines are 14 (Comirnaty), 14 (Spikevax), 37 (Vaxzevria) and 12 (Janssen) per 100,000 doses [20]. Independently of the type of vaccine, the dose number and the causal link, the reports describe the death outcome with a reporting rate of 0.85/100,000 vaccine administrations. The World Health Organization algorithm on vaccine surveillance was applied in 63.4% of reports with a fatal outcome: of these, 59.6% of cases are unrelated, 33.6% are indeterminate, and 4.2% are unclassifiable due to lack of information. Only 2.6% of cases is certainly related to the vaccine [20].

In some European countries (especially in Italy), legal actions against doctors were carried out to ascertain any professional responsibilities of health workers. Some lots of vaccines were seized and inspected. In Italy, in cases in which the patient died following the administration of the vaccine, physicians were investigated for manslaughter. These facts have brought great difficulties to the vaccination campaign for two reasons: the growing distrust of the population and the worries of health professionals. The possibility of being the subject of criminal prosecution for injecting the vaccine creates reluctance on the part of health professionals to work within the vaccination campaign and promote the vaccine. The greatest risk is that of drastically curbing the vaccination campaign.

In Italy in April 2021, in order to avoid the widespread application of a defensive medicine (i.e., the healthcare professional refused to enlist for fear of judicial repercussions), the decree-law 44/2021 was approved. It provides for the exclusion of punishment for healthcare professionals who administer the vaccine if the pharmacovigilance instructions are fully followed and applied. The measure aims to protect the health workers involved in the vaccination campaign, avoiding any criminal liability in the event of adverse effects. In any case, healthcare workers must guarantee correct professional practice: the physicians must collect a complete medical history, must correctly inform the patient about any risks, must carefully evaluate each case, and must avoid administration if there are risk factors for important adverse events. If the adverse event occurs despite compliance with all the indications provided, the doctor is not punishable by criminal law.

However, this provision does not prevent the public prosecutor from carrying out investigations in cases of death following the vaccine to verify the compliance of the inoculation and compliance with all health protocols. In fact, this provision is only a “pro forma” and does not add anything to the rules already in force in Italy. In fact, according to the Italian legislation (Gelli–Bianco law of 2017), if the health professional respects the guidelines, protocols, and health practices of the main administrative authorities (World Health Organization, European Medicines Agency), they will not be able to incur in any kind of criminal danger [21]. The Italian criminal “shield” was only a sign of “closeness” and understanding on the part of the political authorities toward the category of health professionals, but concretely, in the medico-legal practice, nothing has changed.

In any case, from a medico-legal and forensic pathology point of view, all deaths potentially related to the administration of the vaccine must be investigated through a thorough autopsy, histological and immunohistochemical analysis. Only a complete necropsy can allow us to establish a causal link between death and the vaccine, increasing our knowledge of these new vaccines. As a result, risk factors can be established with greater precision, and specific checklists can be drawn up for each vaccine. In this way, these rare adverse events can be reduced even more drastically.

The criminal investigation remains aimed at ascertaining some important aspects: the exhaustive collection of anamnestic data, compliance with international vaccine administration guidelines, and the correct storage and inoculation procedure. The task of the judiciary must run its course in the name of the protection of the population.

## 3. Medical Liability Related to Vaccination: Civil Law Perspective

Several countries in the world provide some form of compensation for injuries or deaths that occur after the administration of a vaccine. Damage compensation is payable, as governments have a responsibility to people impaired by vaccines. Injuries as well as vaccine-related deaths hardly have a clinical, laboratory or autopsy (in the case of death) marker: for this reason, it is not always easy to demonstrate the real cause and the correlation between damage and the vaccine. Causality decisions are generally based on the probability equilibrium standard of “more likely than not” [22].

For example, in the United States, there are two federal compensation systems for people who have been harmed by vaccines: the Countermeasures Injury Compensation Program and the National Vaccine Injury Compensation Program.

In Italy, the law of 25 February 1992, n. 210, recognizes compensation to those irreversibly damaged by vaccinations, transfusions, and the administration of infected blood products.

Compensation is a lump sum amount that is paid to those who have been damaged by vaccination based on the type and severity of the disease and regardless of the ascertainment of medical responsibility. To obtain compensation, it is only necessary to demonstrate the existence of a disease caused by a vaccination.

An important aspect is that bilateral contracts (entered between countries and pharmaceutical companies) provide protection against legal claims [23]. However, for a significant proportion of countries, an indemnity from legal action is constitutionally or financially impossible [23]. Under this scenario, low- and middle-income states could go back to vaccines or could risk having a number of persons harmed by vaccines for whom the government is impotent to offer compensation. Certainly, the lack of compensation to people who are certainly damaged by vaccinations would be a grave social injustice.

To address this issue, Chubb and Marsh (insurance companies) have established a partnership with the World Health Organization and Gavi (The Vaccine Alliance), which guarantees insurance coverage to people in low- to middle-income countries. This program provides an efficient process for receiving compensation for the rare serious adverse events associated with vaccines distributed through COVAX (i.e., distribution of vaccines in poorer countries). This no-fault compensation program is the first international compensation mechanism for damage caused by vaccines.

Across the world, in both rich and poor countries, the creation of a comprehensive system for no-fault compensation for vaccine damage is needed. Excluding countries that are unable to provide compensation would have deprived many people of vaccine protection and would not have allowed them to fight SARS-CoV-2 [23].

To defeat the pandemic, the World Health Organization and all countries of the world must allow quick access to COVID-19 vaccines but also fair insurance coverage that can restore the few cases of vaccine damage.

## 4. Informed Consent

Informed consent is a necessary and essential act in medical practice. In Italy, the obligation for healthcare workers to obtain valid consent from the assisted person is reflected in the Constitution from Article 13 (establishes the inviolability of personal freedom) and Article 32 (no one can be obliged to perform certain health treatments, except by law). It is an integral part of every clinical and surgical intervention [24]. Before implementing a medical and health procedure, the health professional must always fully inform the patient about the benefits, risks and complications and possible alternative procedures.

From an ethical point of view, informed consent should necessarily be considered as an authorization of the patient for vaccination as an autonomous individual. On the other hand, the purpose of informed consent (from an ethical perspective) should be to allow patients to decide (completely autonomously) on the authorization of vaccination.

The role of healthcare professionals is central in the administration of the COVID-19 vaccine: a sterile list of possible complications can make the patient desist from vaccinating. The physician must be able to discuss in depth the benefits of the vaccine (i.e., the development of immunity against SARS-CoV-2), the implications of non-vaccination and the minimal incidence of risks from vaccine. An exhaustive medical interview is necessary to make the patient understand the great preponderance of the benefits over the possible risks. In these cases, correct and complete information is essential. Healthcare professionals play a central role in building trust in vaccines, and their recommendations are important factors in people’s acceptance of the vaccination [25].

On the other hand, physicians must carry out the patient’s medical history, assess their state of health and eligibility for vaccination, considering current and previous pathologies and ongoing therapies.

## 5. Compulsory versus Optional Vaccine in the General Population

In the world, the debate between the fair balance between coercive and persuasive vaccination is always very heated. Some countries have forced vaccination, fueling the issue of the intrusion of the state and health authority in individual freedom and in the parental authority toward their children [1].

Compulsory vaccination can undoubtedly increase the reluctance of a part of the population, especially for poorly informed people. Furthermore, vaccination coercion can discourage healthcare workers from their work to motivate patients to get vaccinated [1].

Because of these problems, some authors [1] believe that compulsory vaccination for the general population should be reserved for catastrophic epidemiological situations. In our opinion, however, this is not quite the case: the population should always put the interest of the community before their own ideas and beliefs. Not getting vaccinated can lead to the onset of COVID-19 outbreaks with a series of consequences: an increase in infections, hospitalizations, and deaths (as well as the social and economic effects due to any generalized closures and lockdowns). This especially endangers people who cannot get vaccinated because of their health or non-responders to vaccination.

In our opinion, therefore, compulsory vaccination cannot be limited only to catastrophic epidemiological situations, but to all those contexts in which free vaccination is not sufficient to achieve collective “herd” immunity. Obviously, if the anamnestic data reveal a high risk of post-vaccination complications, the health professional must avoid the administration or change the type of vaccine.

In fact, the liberty not to get vaccinated compromises the freedom of other people and the community, creating an ethical as well as a legal contrast.

To counter the spread of the virus and the resistance to vaccines of a part of the population, the Italian government has made the so-called “Green pass” to be able to access many events and activities (such as gyms, cinemas, indoor restaurants, museums, theatres, concerts, public competitions, etc.). To obtain the green pass, one must have received at least one dose of the vaccine. The green certification is also issued to those who have obtained a negative result in the molecular/antigen test or recovered from COVID-19. This legislation aims to stimulate the vaccination campaign and to avoid future lockdowns, curfews, or closures of commercial activities.

However, until August 2021, no regulations were issued by the Italian government for the compulsory vaccination of the population.

In Italy, compulsory vaccination must be regulated by a specific law to comply with the second paragraph of Article 32 of the Constitution (“no one can be obliged to a specific health treatment except by law”).

In this regard, the recent sentence 116/2021 of 8 April 2021 of the European Court of Human Rights in Strasbourg analyzed the law of the government of the Czech Republic, which precludes unvaccinated children from enrolling in kindergarten. According to the Court, the law does not violate Article 8 (right to respect for private life) of the European Convention on Human. In fact, the goal is to protect against diseases that can represent a serious health risk. This refers to vulnerable people who are counting on achieving so-called herd immunity. This aim corresponds to the purposes of protecting the health and rights of others, recognized in Article 8. Each state must obtain the right balance between public interest and interference in private life, using the most suitable and appropriate means to achieve an equilibrium.

In any case, in the member states of the convention, there is not a single model on compulsory vaccination. The balance between self-determination and obligation has different nuances in different countries. Some legal systems (such as that in France) provide for the incontrovertible obligation of vaccination, limiting the possibility of choice. On the other hand, there are legal systems (such as that in the United Kingdom) that give greater weight to self-determination. According to the Strasbourg Court, the aim is to protect the health of citizens and protect vulnerable individuals for whom the rest of the population is asked to be vaccinated [26].

The sentence opens the doors to future regulations of the European States that introduce the vaccination obligation, especially in situations where voluntary vaccination does not guarantee mass protection: the social need to protect individual and public health is preponderant over self-determination.

## 6. Vaccine in Health Professionals

Italy is one of the first countries in the world to have imposed COVID-19 vaccination on health professionals, based on decree-law No. 44/2021. Failure to comply with the vaccination (in people who carry out their activities in health facilities, pharmacies, and professional health offices) determines suspension from work that involves a risk of contagion. In fact, all health professionals who do not get vaccinated are suspended from their health function.

On the other hand, in many other European countries, health professionals have the option of choosing whether to get vaccinated or not.

In this regard, we must remember that health professionals have an obligation to prevent the spread of infectious diseases and to minimize the possible consequences of contagion. In this case, mandatory vaccination has the task of preserving the health of patients, preventing the healthcare worker from being the source and vector of contagion in the nosocomial context. In fact, an unvaccinated doctor can contract the infection and can infect their patients. On the other hand, the contagion in this area concerns hospitalized and sick people who can develop serious consequences.

This aspect leads to the dilemma between the right of the healthcare professional to opt for non-vaccination and the right of patients to protect their health (i.e., the right not to be infected by healthcare professionals). According to a medical–legal analysis, the right of patients not to be infected is prevalent, considering the duty of health professionals in safeguarding collective health. From these considerations derives the legitimacy of imposing the vaccination obligation on health professionals, except for any incompatibilities or contraindications. Healthcare professionals should always be guided by the principle of guaranteeing the health of populations and patients.

On the other hand, any reluctance of the health worker to vaccinate obviously determines further instability and skepticism of the population. Patients often rely on their physician, which is why insecurity on the part of health professionals generates considerable distrust. How can a person be persuaded to get vaccinated if their physician does not want to get vaccinated? In this context, therefore, it is necessary that the health workers have faith in science, their work and studies. In fact, an inevitable effect of the health professional’s vaccination is the example it gives in promoting vaccination reliability [1].

It is the ethical responsibility of health professionals to generate trust in vaccines, using scientific knowledge, avoiding the dissemination and promotion of disinformation [1].

To promote understanding of the importance of vaccination, healthcare professionals should use simple, collaborative, and reassuring language. A decision fully shared by the doctor and patient is the best way. In this regard, shared decision making (SDM) includes the involvement of the patient in the healthcare decision-making process. SDM promotes patients’ rights and autonomy and is a strategy for encouraging evidence-based medicine. In this case, the goal is to increase patient confidence in vaccination, addressing vaccine doubts through patient–healthcare worker interaction, including gaps in knowledge, understanding how vaccines work, the disease they prevent, the very low complication rates and risk concepts [27,28].

## 7. Vaccine and Psychological Repercussions

As in other countries [29], in Italy, the interest of scientists is focusing on the psychological reaction of the population to the vaccination campaign, both in its passive/active acceptance and in vaccine hesitance and resistance [30].

The prospect of a return to pre-pandemic “normality” supported by the distress that these two years of distancing and social limitations have entailed [31] are the main factors that, in Italy, are animating adherence to mass vaccination.

Despite this, there are “no-vax” campaigns that are leading the government to develop strategies aimed at containing this attitude (“green pass” mandatory from 6 August 2021 for all citizens over 12 years of age for access social events and activities).

In fact, a recent study by the University of Milan [32] highlighted how the percentage of those against the vaccine, as well as the number of skeptics, decreased significantly from December 2020 to July 2021. At that time, 12% of the sample declared not to be available to undergo vaccination, while 18% said they were skeptical of it. The most recent data show a decrease of 5% of those opposed and 8% of the hesitant ones.

There are several psychological dispositions that traverse personality, cognitive styles, emotion, beliefs, trust, and socio-political attitudes that distinguish those who are hesitant or resistant to a COVID-19 vaccine from those who are accepting [32].

For these reasons, it is essential to deepen and analyze the mechanisms underlying these attitudes, starting from the general theories existing in the literature and then “understanding” the single national reality and local sub-cultures.

Recently, some authors provided a novel tool to determine psychological antecedents concerning the vaccination decision named the “5C scale”. Published in 2018, the 5C scale is psychometrically validated to assess five psychological antecedents of vaccination [33].

In brief, the 5C scale aims to assess the following determinants: (1) confidence in the safety and efficacy of vaccines and trust in the providers, including policymakers and healthcare workers providing the service; (2) complacency, which is defined by the low-perception of disease risk; (3) constraints, which include the physical and psychological barriers rendering vaccination inconvenient; (4) calculation, which entails active engagement in searching for information about the vaccine and its utility; and (5) collective responsibility, defined by the extent of willingness to benefit others by receiving vaccination to help in achieving herd immunity. [34].

This tool is proving to have good discriminatory power to predict COVID-19 vaccines antecedents among national reality and local sub-cultures. [35,36].

Only in this way can we develop communicative intervention strategies to provide the right scientific information.

All this has even more significance considering the negative impact that the pandemic is having on mental health in general and, above all, on young people [37].

## 8. Conclusions

Humans have a long history of fighting viruses, and the defeat of serious diseases has been possible only thanks to vaccines. The SARS-CoV-2 pandemic has caused serious repercussions in the clinical and health fields but also in forensic pathology [38,39,40,41]. The global vaccination campaign is involving several medico-legal debates.

The end of the pandemic will only be possible after complete vaccination around the world. Health professionals are tasked with promoting confidence in vaccination for the general population by making them understand the preponderance of benefits over minimum risks. Complete information and reliance on scientific research are essential to understand the great importance of the vaccination campaign.

From a criminal point of view, we must avoid blaming the health professionals in the case of side effects. At the same time, we must protect the population, ensuring compliance with the indications, guidelines, and an adequate method of administration. On the other hand, from a civil law perspective, it is right to ensure full protection for those rare cases in which the administration of the vaccine is related to adverse events.

No drug is totally free from possible complications and unforeseen events: it is possible that in the future, there will be other cases of adverse events, but without a global vaccination campaign, it will be impossible to defeat the virus.

The impact that the pandemic is having on youth mental health must be an additional stimulus to overcome hesitation and opposition to the vaccination campaign by planning communicative interventions that strengthen trust in vaccines.

Finally, from a forensic pathology point of view, all fatal adverse events following the vaccine must be investigated in depth. The autopsy investigation can allow a better understanding of the pathophysiological process. This can lead to a greater definition of risk factors and, consequently, a decrease in the incidence of these events.

In conclusion, the aim of this article is to discuss the issues and, at the same time, the importance of mass vaccination. The end of the pandemic will be made possible through global vaccination, capable of minimizing hospitalizations from SARS-CoV-2 infection. If through a “voluntary” vaccination, mass protection is not achieved, “compulsory” vaccination (imposed by governments) will undoubtedly be necessary.

## Data Availability

For the data we refer to the citations.
